# Wavenumber-Domain Joint Estimation of Rotation Parameters and Scene Center Offset for Large-Angle ISAR Cross-Range Scaling

**DOI:** 10.3390/s25113444

**Published:** 2025-05-30

**Authors:** Bakun Zhu, Weigang Zhu, Hongfeng Pang, Chenxuan Li, Lei Qui, Jinhai Yan, Fanyin Ma, Yijia Liu

**Affiliations:** 1 Department of Electronic and Optical Engineering, Space Engineering University, Beijing 101416, China; 2 Beijing Aerospace Command and Control Center, Beijing 100094, China

**Keywords:** inverse synthetic aperture radar (ISAR) imaging, cross-range scaling, non-uniform rotation, wavenumber domain, scene center offset (SCO)

## Abstract

While the wavenumber-domain approach enables large-angle inverse synthetic aperture radar (ISAR) cross-range scaling, its practical application remains constrained by the target’s non-uniform rotation and scene center offset (SCO). In response to this issue, this paper introduces a novel large-angle ISAR cross-range scaling method through a joint estimation method based on the wavenumber domain. A non-uniform rotational wavenumber-domain signal model with SCO is developed. Utilizing this model and the sensitivity of wavenumber-domain imaging to SCO, a joint estimation algorithm that combines particle swarm optimization (PSO) and image entropy evaluation is proposed, achieving accurate parameter estimation. Leveraging the estimated parameters, the range and cross-range scaling factors in the wavenumber-domain imaging are derived, facilitating ISAR cross-range scaling with higher accuracy than the traditional method. The effectiveness and robustness of the proposed method are validated under various conditions, through scattering point and electromagnetic computing simulation.

## 1. Introduction

Inverse synthetic aperture radar (ISAR) imaging is a pivotal technology for acquiring two-dimensional images of targets, providing high-resolution capabilities independent of detection distance and robust performance under all-weather conditions and finding extensive applications in target detection and environmental monitoring [1,2]. ISAR images, reflecting the two-dimensional electromagnetic scattering characteristics of targets, serve as a vital source of features for automatic target recognition (ATR) engineering [3]. However, these images represent mere projections onto the range–Doppler (RD) plane, where the Doppler dimension may not accurately depict the actual size of the target, thereby complicating feature extraction and ATR processes [4,5]. To address this, cross-range scaling techniques are essential for producing ISAR images with accurate target dimensions.

In ISAR imaging, the resolution along the range direction is primarily governed by the bandwidth of the transmitted signal. Conversely, the resolution in the cross-range direction is influenced by both the wavelength of the transmitted signal and the rotation parameters of the target [6]. In most cases, determining target rotation parameters poses a significant challenge, complicating the process of cross-range scaling in ISAR images. This scaling process typically involves two primary steps: initially estimating the target’s rotation parameters and subsequently correlating these parameters with the cross-range scaling factors to achieve accurate image scaling. The scaling methodologies can be classified into three distinct categories based on their underlying principles.

The first method, known as the rotation correlation method, leverages the rotational motion of the target captured across different sub-aperture ISAR frames. By analyzing the correlation between these rotated sub-aperture images [7], or the prominent scatter points within them [8,9], the rotation angle between consecutive imaging instances can be deduced. Furthermore, the target’s rotation angular velocity (RAV) can be calculated. An adaptation of this method involves implementing rotational correlation within the polar coordinate domain to estimate RAV [10]. Although the rotation correlation method allows for a direct estimation of the rotation angle, it demands a high-quality ISAR image and yields lower accuracy in parameter estimation.

The second method, phase coefficient estimation, constructs the phase history of scatter points as a second- or higher-order polynomial in slow time. This method establishes a linear relationship between the second-order phase coefficients and the scatter points’ range position to estimate the target’s RAV. The accuracy of phase coefficient estimation is crucial as it directly impacts the precision of cross-range scaling. Consequently, the modified Wigner–Ville distribution [11] and LVD transformation [12] have been applied to the estimation of second-order phase coefficients to enhance the estimation accuracy. Additionally, the integration of local polynomial Fourier transformation (LPFT) with least squares estimation [13] and the use of 2D discrete wavelet transform combined with pseudopolar Fourier transform [14] have been employed to improve algorithm robustness. The phase history model based on a second-order polynomial is applicable solely to uniformly rotating targets with limited rotation angles. In the case of a non-uniformly rotating or larger angle, the estimation of RAV is achieved by constructing a higher-order polynomial of phase history and subsequently estimating the phase coefficients [15,16]. Notably, ref. [16] categorizes targets into stationary and maneuvering types. For maneuvering targets, the linear component of the chirp rate is estimated using the integral of Lv’s distribution; however, constant and linear chirp rates are limited to scenarios involving small rotation angles. For large-angle scenarios, it is challenging to perform imaging based on the RD principle. Furthermore, even if imaging can be achieved and high-order phase coefficients are accurately estimated, establishing a correspondence between these phase coefficients and rotational parameters remains difficult. Severe range cell migration caused by a large rotation angle will lead to an extremely complex polynomial phase signal in a range cell. Additionally, by employing range instantaneous Doppler (RID) imaging to reduce imaging coherent processing intervals (CPIs), scaling is performed using the instantaneous RAV [17,18,19]. Nevertheless, the inherent cross-terms in RID leads to a degradation in imaging quality, and the scaling is similarly grounded in the estimation of phase coefficients. In essence, RID mitigates defocusing during imaging by reducing the CPI, which conflicts with the principle of large-angle imaging that enhances resolution through CPI extension. Therefore, RID-based scaling methods are unsuitable for large-angle scenarios.

The third category involves image quality optimization, which formulates an optimization model by exploring the correlation between target rotation parameters and image quality [20]. This approach yields optimal rotation parameters through techniques such as the Gauss–Newton method [21,22,23] and Particle Swarm Optimization (PSO) [24]. Specifically, [22] enhances the Hessian matrix within the Newton method to facilitate rapid convergence of the estimation algorithm, although this method is limited to uniformly rotating targets. Subsequently, [23] extends the model to accommodate non-uniform rotation scenarios over extended CPIs. Reference [24] integrates interpolation and keystone processes to augment image quality, applying PSO for parameter optimization. Image quality optimization methods often exhibit good noise robustness. However, since these methods are based on the range–Doppler (RD) principle for signal modeling, the constructed optimization models are typically constrained by strong scenario dependencies, limiting their applicability to targets with specific rotation angles and non-uniform rotation levels. For large-angle imaging, continued reliance on RD-based signal modeling significantly increases model complexity. Reference [25] employs the Polar Format Algorithm (PFA) for ISAR imaging, where rotation estimation parameters are obtained through a Monte Carlo search. By establishing a large-angle signal model in the wavenumber domain, this approach substantially reduces subsequent optimization model complexity. Nevertheless, parameter estimation accuracy degrades under non-uniform rotation and scene center offset (SCO) conditions.

The traditional PFA relies on two critical assumptions: uniform target rotation and coincidence between the rotation center and scene center. The violation of these assumptions degrades PFA imaging performance. Therefore, this study adopts wavenumber-domain signal modeling and employs the image quality optimization method to estimate the non-uniform rotation parameters and SCO, thereby achieving high-precision scaling. The main contributions of this study are as follows:

(1) A wavenumber-domain signal model incorporating SCO for large-angle ISAR imaging of non-uniformly rotating targets is introduced. Unlike the traditional PFA, which typically neglects non-uniform target rotation and SCO, which cause degradation in large-angle imaging and reduced scaling accuracy, the new wavenumber-domain model explicitly accounts for these adverse effects, enabling more precise characterization of target rotation. In addition, compared with conventional RD imaging models, the wavenumber-domain signal model demonstrates enhanced conciseness and efficacy.

(2) A scaling method based on the joint estimation of non-uniform rotation parameters and SCO is developed. Leveraging the inherent sensitivity of wavenumber-domain imaging to both non-uniform rotation and SCO, we formulate an image-quality-driven optimization model for parameter estimation. Following optimal parameter acquisition through PSO searching, accurate scaling for large-angle imaging across diverse scenarios is achieved using derived wavenumber-domain scaling factors.

The remainder of this paper is organized as follows: Section 2 introduces the ISAR imaging signal model for non-uniformly rotating targets. Section 3 systematically elaborates on the proposed cross-range scaling methods, including the joint estimation method for rotation parameters and SCO, as well as the wavenumber-domain cross-range scaling method. Section 4 validates the scaling accuracy and robustness of the proposed methods using simulation data. Section 5 concludes the paper.

## 2. ISAR Signal Model

In ISAR imaging, high range resolution is achieved by transmitting signals with large bandwidths, while Doppler resolution is obtained through the accumulation of multi-aspect pulses within the CPI. The formation of Doppler resolution in ISAR imaging is closely related to the target’s motion. Assuming the target is composed of P scattering points and the slow time is $t_{m}$, the distance from each scattering point to the radar can be denoted as $R_{rp}(t_{m})$, which reflects the relative motion between the radar and the target.

Assuming the radar transmits linear frequency modulated (LFM) signals and utilizes dechirp for reception, the radar echo after pulse compression can be represented as follows:

(1) $$s_{r}\left(\hat{t};t_{m}\right)=\sum_{p=1}^{P}\sigma_{p}\mathrm{sinc}\left[B\cdot \left(\hat{t}-\frac{2R_{rp}\left(t_{m}\right)}{c}\right)\right]\exp\left[-j2\pi f_{c}\frac{2R_{rp}\left(t_{m}\right)}{c}\right]$$

where $\sigma_{p}$ denotes the reflection coefficient of the scattering point, B is the signal bandwidth, t represents the fast time, c is the speed of light, and $f_{c}$ is the carrier frequency.

**Figure 1:**
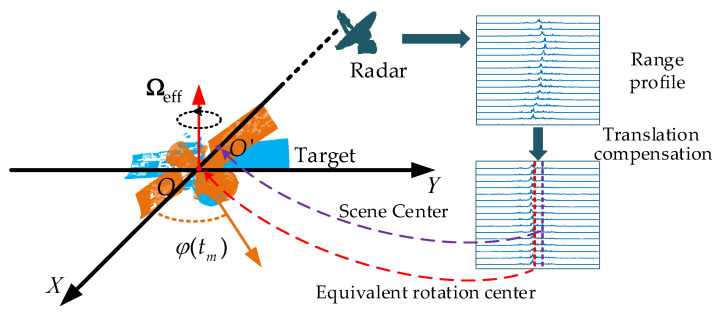
Illustration of ISAR imaging geometry and SCO.

$R_{rp}\left(t_{m}\right)$ encompasses both translational and rotational motion components. The rotational motion is crucial for imaging, while the translational motion typically leads to a loss of coherence among the echoes from different pulses. Thus, translational compensation is necessary to restore coherence. Figure 1 illustrates the ISAR imaging geometry and SCO. In the ideal scenario of translational motion compensation, the radar echoes are equivalent to those produced by the target rotating around the equivalent rotation center (ERC) O, where $\Omega_{\mathrm{eff}}$ represents the angular velocity vector. In the Cartesian coordinate system XOY, where the X-axis is aligned with the radar line of sight (LOS) and the coordinate plane is perpendicular to $\Omega_{\mathrm{eff}}$, the non-uniform rotation of the target around the equivalent rotation center can be represented by an angle, denoted as $\varphi \left(t_{m}\right)$.

The image center after ISAR imaging is designated as the scene center, denoted as point O’ in Figure 1. Since the ERC position depends on multiple factors including translational motion compensation methods, electromagnetic scattering characteristics of the target, and motion states, the scene center typically deviates from the ERC. Since translational motion compensation addresses the slow-time-dependent motion components, the SCO manifests as a constant-term translational distance in radar echoes, which appears as target displacement along the range direction in RD imaging. Considering the SCO, under far-field conditions, the echo signal after translational motion compensation [26,27,28,29] can be expressed as

(2) $$s_{rp}\left(\hat{t};t_{m}\right)=\sum_{p=1}^{P}\sigma_{p}\mathrm{sinc}\left[B\cdot \left(\hat{t}-2\frac{x_{p}\cos\varphi (t_{m})+y_{p}\sin\varphi (t_{m})-△x}{c}\right)\right]\exp\left[-j4\pi f_{c}\frac{x_{p}\cos\varphi (t_{m})+y_{p}\sin\varphi (t_{m})-△x}{c}\right]$$

where $\left[x_{p},y_{p}\right]$ represents the position of the scattering point in the coordinate system XOY and △x is the SCO. △x induces a range shift in RD imaging results but does not compromise image quality. For wavenumber-domain imaging, however, △x not only causes positional offsets but also defocuses images (This phenomenon will be presented in Section 4.2). Consequently, △x must be incorporated into the signal model to ensure accuracy.

In the classical turntable model [30], $\varphi (t_{m})$ is a linear variable. However, in practical scenarios such as spacecraft target imaging, as the imaging CPI increases [23] and the target maneuverability is enhanced [16], a linear variable is insufficient to describe the non-uniform rotation of the target. Therefore, $\varphi (t_{m})$ is modeled as a quadratic polynomial with respect to slow time:

(3) $$\varphi (t_{m})=\omega t_{m}+\alpha t_{m}^{2}$$

where ω is the RAV and α is the rotation angular acceleration (RAA).

By expanding the trigonometric functions in Equation (2), the radar echo signal can be further expressed as

(4) $$s_{r}\left(\hat{t};t_{m}\right)=\sum_{p=1}^{P}\sigma_{p}\mathrm{sinc}\left[B\cdot \left(\hat{t}-2\frac{x_{p}-△x+y_{p}\omega t_{m}-\frac{x_{p}\omega^{2}t_{m}^{2}}{2}+C_{oth}}{c}\right)\right]\exp\left[-j4\pi f_{c}\frac{x_{p}-△x+y_{p}\omega t_{m}-\frac{x_{p}\omega^{2}t_{m}^{2}}{2}+C_{oth}}{c}\right]$$

where $C_{oth}$ represents other polynomials in the Taylor expansion, which can be further expanded as

(5) $$C_{oth}=y_{p}\alpha t_{m}^{2}-x_{p}\omega \alpha t_{m}^{3}-\frac{y_{p}\omega^{3}t_{m}^{3}}{6}+\ldots .$$

In Equation (4), $y_{p}\omega t_{m}-0.5x_{p}\omega^{2}+C_{oth}$ is associated with range migration. Under large rotation angles, it easily exceeds one range cell, resulting in range migration. This highlights the difference between large-angle imaging and conventional RD imaging, as severe range cell migration can compromise the phase coefficient model. Then, errors will be introduced in the estimation of rotation parameters when phase coefficients are extracted from the phase history. Moreover, most cross-range methods estimate the second-order phase coefficient $x_{p}\omega^{2}/2$ and subsequently estimate the RAV. When the target undergoes non-uniform or large-angle rotation, $C_{\mathrm{oth}}$ will affect the estimation of the second-order phase coefficient, causing a decline in cross-range scaling accuracy (These effects will be presented in Section 4.1 and Section 4.3). Even with advanced time-frequency analysis methods capable of estimating high-order phase coefficients in $C_{\mathrm{oth}}$, the specific correspondence between these high-order terms and particular components within $C_{\mathrm{oth}}$ remains undetermined. Such correspondence depends on the concrete imaging scenario. Failure to establish the relationship between high-order phase coefficients and motion parameters will compromise scaling accuracy or lead to failed scaling.

## 3. Algorithm Description

According to the analysis presented in Section 2, the phase history polynomial model reveals inaccuracies when describing targets with complex rotation. Consequently, the methods for estimating rotational parameters and cross-range scaling based on this model are subject to strict applicability conditions, leading to diminished algorithm performance, particularly in large-angle imaging scenarios. In response, Section 3 develops an alternative approach by modeling the signal from the wavenumber-domain perspective. This approach aims to construct a cross-range scaling method that is more adaptable to large-angle imaging. Section 3 is structured as follows: Section 3.1 discusses joint methods for estimating rotational parameters and SCO, Section 3.2 investigates precise scaling methods within the wavenumber domain, and Section 3.3 outlines the comprehensive process for the cross-range scaling algorithm.

### 3.1. Joint Estimation of Rotation Parameters and SCO

The PFA focuses ISAR imaging by transforming the echo data from the range–slow time domain to the wavenumber domain, effectively decoupling the range frequency from the slow time. Compared with the phase history polynomial model, the wavenumber-domain signal model offers a simpler formulation and demonstrates suitability for large-angle rotation scenarios. However, the PFA requires precise knowledge of rotational parameters and strict alignment between the ERC and the scene center. Inaccurate rotational parameter estimation or SCO degrades imaging performance. By leveraging these challenges, a combined approach of image quality optimization and wavenumber-domain imaging can facilitate high-precision estimation of rotational parameters and SCO.

Applying an inverse Fourier transform along the fast time to Equation (2) results in

(6) $$s(f_{r},t_{m})=\frac{\pi }{B}\sum_{p=1}^{P}\sigma_{p}rect\left(\frac{f_{r}}{B}\right)\exp\left[-j4\pi \left(f_{c}+f_{r}\right)\frac{x_{p}\cos\varphi (t_{m})+y_{p}\sin\varphi (t_{m})}{c}\right]\exp\left[-j4\pi \left(f_{c}+f_{r}\right)\frac{-△x}{c}\right]$$

where $f_{r}$ denotes range frequency. If the accurate estimated values of △x are known, compensation terms can be constructed to eliminate SCO, expressed as

(7) $$\begin{array}{l} s_{c}(f_{r},t_{m})=s(f_{r},t_{m})\exp\left[-j4\pi \left(f_{c}+f_{r}\right)\frac{△\hat{x}}{c}\right]\\ \;\;\;\;\;=\frac{\pi }{B}\sum_{p=1}^{P}\sigma_{p}rect\left(\frac{f_{r}}{B}\right)\exp\left[-j4\pi \left(f_{c}+f_{r}\right)\frac{x_{p}\cos\varphi (t_{m})+y_{p}\sin\varphi (t_{m})}{c}\right] \end{array}$$

Transforming the echo generation in Equation (7) from a sequential accumulation of scattering points to a two-dimensional integral, and omitting constants that do not affect imaging leads to

(8) $$s_{c}(f_{r},t_{m})\;\;=\iint \sigma (x,y)rect\left(\frac{f_{r}}{B}\right)\exp\left[-j4\pi \left(f_{c}+f_{r}\right)\frac{x_{p}\cos\varphi (t_{m})+y_{p}\sin\varphi (t_{m})}{c}\right]dxdy$$

where σ(x,y) is the scattering point intensity at position [x,y].

The relationship between the range frequency–slow time domain and the wavenumber domain is defined by

(9) $$\left\{\begin{array}{l} f_{x}=\frac{2\left(f_{c}+f_{r}\right)\cos\varphi (t_{m})}{c}\\ f_{y}=\frac{2\left(f_{c}+f_{r}\right)\sin\varphi (t_{m})}{c} \end{array}\right.$$

where $f_{x}$ and $f_{y}$ represent the spatial frequencies in the wavenumber domain.

**Figure 2:**
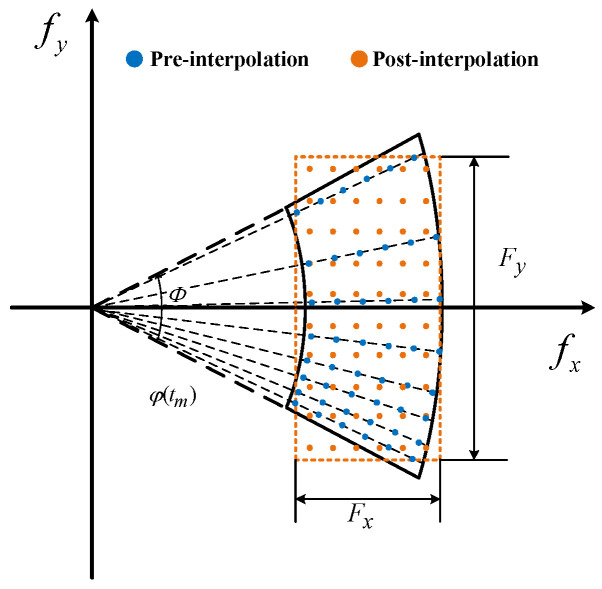
The illustration of wavenumber-domain interpolation.

With $\varphi (t_{m})$ known, by combining Equations (8) and (9), we can obtain the wavenumber-domain signal after coordinate transformation and interpolation [31].

(10) $$s_{c}(f_{x},f_{y})=rect\left(\frac{f_{x}-2f_{c}/c}{F_{x}}\right)rect(\frac{f_{y}}{F_{y}})\iint \sigma (x,y)\;\;\exp\left[-j2\pi \left(xf_{x}+yf_{y}\right)\right]dxdy$$

where $F_{x}$ and $F_{y}$ represent the distribution ranges of the interpolated data in the wavenumber domain. The interpolation process can be illustrated as shown in Figure 2.

In Figure 2, the effective rotation angle (ERA) of the LOS during the imaging CPI is denoted as Φ. Direct conversion of radar echo data to the wavenumber domain results in the non-uniform distribution of data points (shown in blue), necessitating further interpolation to achieve uniformity (shown in orange). To encompass all the original wavenumber-domain data, this paper constrains the wavenumber range of the interpolated data based on the minimum and maximum values of the pre-interpolation data distribution in the $f_{x}$ and $f_{y}$ directions. Then, the variables $F_{x}$ and $F_{y}$ can be represented as follows:

(11) $$\begin{array}{l} F_{x}=\frac{f_{\max}\max\left[\cos\left(\hat{\varphi }(t_{m})\right)\right]}{c}-\frac{f_{\min}\min\left[\cos\left(\hat{\varphi }(t_{m})\right)\right]}{c}\\ F_{y}=\frac{f_{\max}\max\left[\sin\left(\hat{\varphi }(t_{m})\right)\right]}{c}-\frac{f_{\min}\min\left[\sin\left(\hat{\varphi }(t_{m})\right)\right]}{c} \end{array}$$

where $f_{\min}$ and $f_{\max}$ are the maximum and minimum values of the range frequency.

Performing a 2D-IFT on Equation (10) yields the ISAR image.

(12) $$s_{\mathrm{Img}}(\hat{\omega },\hat{\alpha },△\hat{x})=2D-\mathrm{IFT}\left[s_{c}(f_{x},f_{y})\right]$$

where the imaging result is directly determined by the estimated values of rotational parameters and SCO; thus, the ISAR image can be denoted as $s_{\mathrm{Img}}(\hat{\omega },\hat{\alpha },△\hat{x})$.

As a well-established image quality evaluation metric, IE has been widely applied to ISAR parameter estimation [22] and autofocus imaging [32]. Therefore, to optimize the image quality, we employ IE to evaluate the imaging result, which is defined as

(13) $$\left\{\begin{array}{l} IE\left(s_{\mathrm{Img}}\right)=\ln E_{s}-\frac{1}{E_{s}}\sum_{m=1}^{M}\sum_{n=1}^{N}A_{mn}\cdot \ln(A_{mn})\\ A_{mn}={\left|g_{mn}\right|}^{2}\\ E_{s}=\sum_{m=1}^{M}\sum_{n=1}^{N}A_{mn}\cdot \ln(A_{mn}) \end{array}\right.$$

where $g_{mn}$ is the magnitude of the pixel in the ISAR image. The indices m and n are the coordinates for each pixel. M and N denote the number of pixels in each dimension.

Through the described analysis, we have developed a joint estimation optimization model for rotational parameters and SCO.

(14) $$[\hat{\omega },\hat{\alpha },△\hat{x}]=\arg\min IE\left(s_{\mathrm{Img}}(\hat{\omega },\hat{\alpha },△\hat{x})\right).$$

When $\hat{\omega }$, $\hat{\alpha }$, and $△\hat{x}$ are accurately estimated, the ISAR image exhibits minimal IE, indicating the optimal imaging quality.

**Figure 3:**
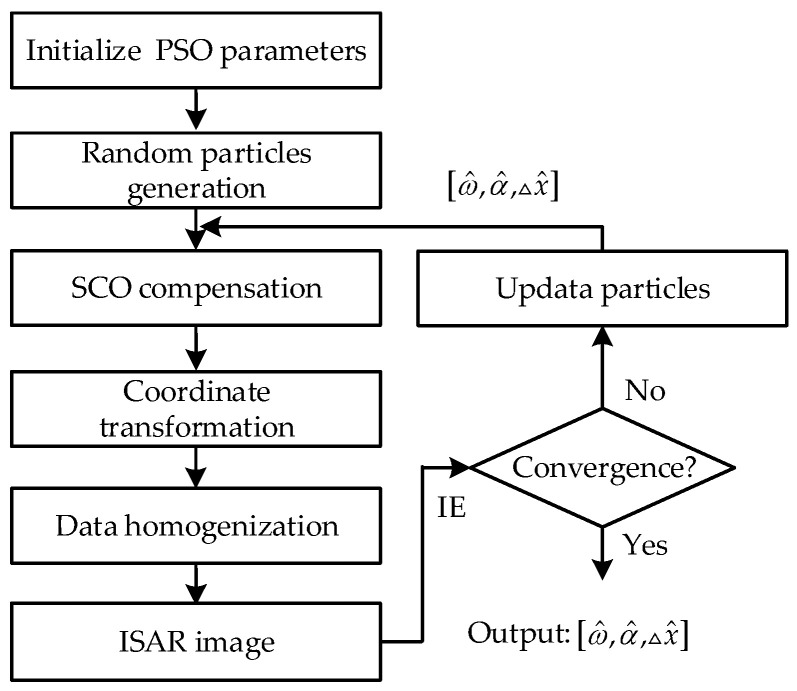
Flowchart of the joint estimation algorithm for non-uniform rotation parameters and SCO based on the wavenumber domain.

Due to the complexity of coordinate transformations and interpolation, deriving IE with respect to the estimated parameters presents challenges. To address this, an intelligent optimization algorithm, specifically PSO, is employed for its rapid convergence and robust global optimization capabilities [33]. PSO is thus integral to our model, searching for the optimal estimated parameters. Figure 3 illustrates the flowchart of the joint estimation algorithm for non-uniform rotation parameters and SCO in the wavenumber domain, with specific steps outlined as follows.

Step 1: Initialize the parameters of the PSO algorithm. Among these, the initial parameter ranges and the number of particles is crucial for the algorithm’s optimization and convergence. The range of SCO is set to ±0.5 times the range dimension of the ISAR image. The ranges of RAV and RAA can be reasonably configured based on the imaging scenario. If the imaging scenario is completely unknown, a larger initial range for RAV and RAA can be set with a small number of particles (e.g., three particles) for exploration. The search ranges for RAV and RAA can then be narrowed, and the number of particles can be increased based on the search results. In the scenario of this study, satisfactory accuracy can be achieved when the number of particles reaches 10.

Step 2: Generate particles randomly, each representing a three-dimensional vector of the rotation parameters and estimated SCO.

Step 3: For each particle, construct the SCO compensation term based on the estimated parameters and compensate the echo signal, as described in Equation (7).

Step 4: Convert the SCO-compensated echo data to the wavenumber domain per Equation (6) and apply bilinear interpolation to ensure uniform data distribution. The interpolation range is determined based on Equation (11).

Step 5: Execute a 2D inverse Fourier transform (2D-IFT) on the interpolated echo data to obtain the ISAR image, and compute the IE as per Equation (13).

Step 6: Assess convergence based on the IE results. If convergence is achieved, the parameters $\left[\hat{\omega },\hat{\alpha },△\hat{x}\right]$ are finalized; otherwise, update the particles and repeat Steps 2–6.

By the algorithm, non-uniform rotation parameters and SCO can be obtained, facilitating ISAR imaging and cross-range scaling.

### 3.2. Cross-Range in the Wavenumber Domain

The ISAR imaging results, as derived from Equation (12), initially delineate the shape and scattering points distribution characteristics of a target, albeit without providing the target’s actual physical dimensions. To ascertain these dimensions, scaling is essential, which involves computing the actual physical length represented by each pixel unit.

Based on Equation (10), we consider the wavenumber-domain echo of a single scattering point, represented as

(15) $$s_{cp}(f_{x},f_{y})=rect\left(\frac{f_{x}-2f_{c}/c}{F_{x}}\right)rect(\frac{f_{y}}{F_{y}})\sigma (x_{p},y_{p})\exp\left[-j2\pi \left(x_{p}f_{x}+y_{p}f_{y}\right)\right].$$

Discretization is performed on the echo.

(16) $$s_{cp}(n_{x},n_{y})=rect\left(\frac{n_{x}△f_{x}-2f_{c}/c}{N_{x}△f_{x}}\right)rect(\frac{n_{y}△f_{y}}{N_{y}△f_{y}})\sigma (x_{p},y_{p})\exp\left[-j2\pi \left(x_{p}n_{x}△f_{x}+y_{p}n_{y}△f_{y}\right)\right]$$

where $△f_{x}$ and $△f_{y}$ represent the discrete spatial frequency units for $f_{x}$ and $f_{y}$, respectively, with $N_{x}$ and $N_{y}$ as the discrete quantities $n_{x}$ and $n_{y}$ as the indices of these discrete units.

By performing the discrete inverse Fourier transform (2D-DIFT) on Equation (16), the complex signal representation of the scattering point in the image domain can be obtained as

(17) $$\begin{array}{c} s_{\mathrm{Img},p}(k_{x},k_{y})=\frac{1}{N_{x}N_{y}}\sum_{n_{x}=0}^{N_{x}-1}\sum_{n_{y}=0}^{N_{y}-1}s_{cp}(n_{x},n_{y})\;\exp\left[j2\pi \left(\frac{n_{x}k_{x}}{N_{x}}+\frac{n_{y}k_{y}}{N_{y}}\right)\right]\;\\ \;\;\;\;\;\;=\frac{\sin\left(\pi \left(k_{x}-2x_{p}F_{x}\right)\right)}{N_{x}\sin\left(\pi \left(k_{x}-2x_{p}F_{x}\right)/N_{x}\right)}\frac{\sin\left(\pi \left(k_{y}-2y_{p}F_{y}\right)\right)}{N_{y}\sin\left(\pi \left(k_{y}-2y_{p}F_{y}\right)/N_{y}\right)}\exp\left[j2\pi \frac{k_{x}f_{c}}{cF_{x}}\right]\exp\left[-j4\pi \frac{x_{p}f_{c}}{c}\right]\sigma (x_{p},y_{p}) \end{array}$$

where $k_{x}$ and $k_{y}$ denote the discrete indices in the two-dimensional image domain. The detailed derivation of Equation (17) can be found in the Appendix A.

From Equation (17), it is evident that the peak position of the scattering point $\left[x_{p},y_{p}\right]$ in the image is

(18) $$\left\{\begin{array}{l} k_{x}=2x_{p}F_{x}\\ k_{y}=2y_{p}F_{y} \end{array}\right.$$

Further, the calculation formulas of the actual physical position of the scatter points can be derived.

(19) $$\left\{\begin{array}{l} x_{p}=\varsigma_{r}k_{x}\\ y_{p}=\varsigma_{c}k_{y}\\ \varsigma_{r}=\frac{1}{2F_{x}}\\ \varsigma_{c}=\frac{1}{2F_{y}} \end{array}\right.$$

where $\varsigma_{r}$ and $\varsigma_{c}$ are the range and cross-range scaling factors, respectively, which are directly determined by $F_{x}$ and $F_{y}$.

Equation (11) provides the expressions of $F_{x}$ and $F_{y}$, within which the frequency of the signal can be represented as

(20) $$f=f_{r}+f_{c}.$$

where the range frequency $f_{r}$ is centered at 0 Hz with a bandwidth equal to the radar bandwidth. Therefore,

(21) $$\left\{\begin{array}{l} f_{\min}=f_{c}-\frac{B}{2}\\ f_{\max}=f_{c}+\frac{B}{2}. \end{array}\right.$$

According to Figure 2, the ERA Φ that the radar LOS rotates is given by

(22) $$\Phi =\hat{\varphi }(t_{M})-\hat{\varphi }(t_{0})$$

where $t_{M}$ and $t_{0}$ represent the end and start times of the slow time, respectively.

Combining Equations (11), (19), (21) and (22), the scaling factors can be expressed as

(23) $$\left\{\begin{array}{l} \varsigma_{r}=\frac{c}{B\left(1+\cos\left(\frac{\Phi }{2}\right)\right)+2f_{c}\left(1-\cos\left(\frac{\Phi }{2}\right)\right)}\\ \varsigma_{c}=\frac{c}{4f_{c}\sin\left(\frac{\Phi }{2}\right)} \end{array}\right.$$

With scaling factors from Equation (23), accurate scaling for the wavenumber-domain ISAR image can be achieved, thereby allowing for target’s physical dimensions.

### 3.3. Overall Structure of the Algorithm

**Figure 4:**
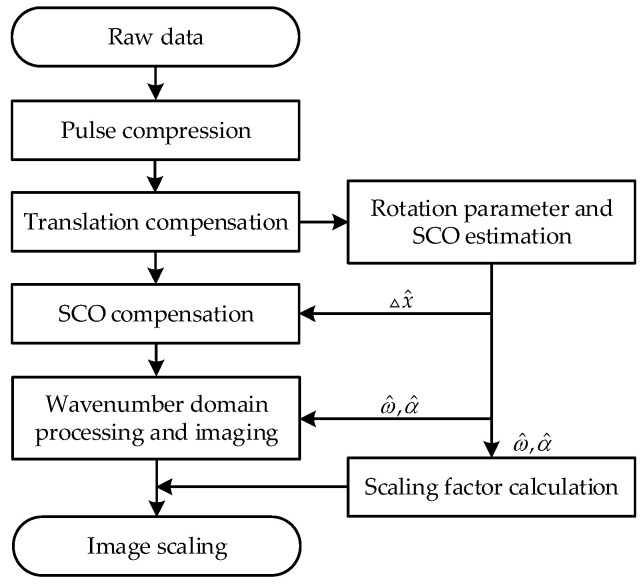
Flowchart of the proposed method.

The flowchart of the proposed method is depicted in Figure 4. Initially, the raw data undergo pulse compression to obtain the range profile, facilitating translational compensation of the radar echo. Subsequently, based on the compensated echo data, non-uniform rotation parameters and SCO are estimated. Following this, SCO compensation and wavenumber-domain processing are conducted, based on the estimated parameters, to obtain a focused ISAR image. Finally, using the estimated non-uniform rotation parameters, the scaling factor is calculated, thereby completing the ISAR image scaling.

Accurate parameter estimation is imperative for the scaling method. The wavenumber-domain model offers a more accurate description of the target rotation in ISAR imaging compared to polynomial phase models and is adept at large-angle imaging scenarios, enhancing the accuracy and applicability of the parameter estimation method. Additionally, the consideration of SCO in wavenumber-domain parameter estimation takes advantage of the characteristic that SCO reduces wavenumber-domain imaging quality, optimizing the parameter estimation for practical imaging processing. Moreover, parameter optimization searches based on PSO and IE ensure robustness and convergence by leveraging the global two-dimensional coherence of radar echo data.

**Figure 5:**
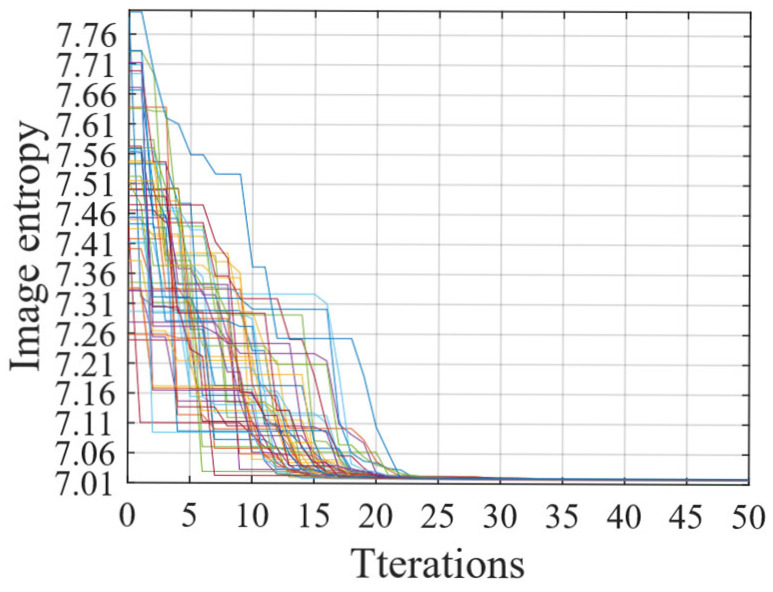
Convergence behavior of the proposed algorithm. (Each colorful curve represents the variation of image entropy across the number of iterations in a simulation, taken from the 50 Monte Carlo simulations discussed in Section 4.4).

The computational complexity of the proposed method can be estimated through the number of complex multiplications involved. Suppose M represents the number of pulses and N the number of range samples. In PSO, each iteration involves $N_{0}$ particles, across a total of $N_{p}$ iterations. The computational load for SCO compensation is O(MN). Using bilinear interpolation to obtain a single data point requires approximately 24 complex multiplications; thus, the computational load for interpolating the entire data matrix becomes O(24MN), which represents the heaviest computational load in the entire processing procedure. And wavenumber-domain imaging using 2D-IFT is $O(MN\log_{2}MN)$. Thus, the overall computational complexity of the proposed method is $O(N_{0}N_{P}(25MN+MN\log_{2}MN))$. Here, $N_{0}$ and $N_{p}$ directly determine the computational load of the algorithm. Considering the limited number of estimated parameters, $N_{0}$ does not need to be set to a high value to ensure algorithm convergence [33]. For $N_{p}$, Figure 5 depicts the algorithmic convergence from 50 Monte Carlo simulations discussed in Section 4.4. It can be observed that the algorithm converges rapidly, ensuring convergence within 30 iterations. Although the algorithm employs heuristic approach and includes interpolation with a heavy computational load, it benefits from reasonable model settings, a small number of estimated parameters, and a straightforward process, resulting in rapid convergence.

In this section, based on the wavenumber-domain imaging model, a joint estimation algorithm for non-uniform rotation parameters and SCO was designed, the wavenumber-domain scaling factor was derived, and the detailed process of the cross-range scaling method was provided, along with a theoretical analysis of computational complexity. The next section presents the validation of the proposed algorithm’s performance through simulations.

## 4. Simulation Study

This section presents scattering point simulation and electromagnetic computation data to validate the efficacy and robustness of the proposed algorithm. In Section 4.1, the merits of wavenumber-domain signal modeling in ISAR imaging of large rotation angles are demonstrated. Section 4.2 and Section 4.3 investigate the effects of the SCO and non-uniform rotation on the scaling methods, respectively. In Section 4.4, the scaling performance under concurrent SCO and non-uniform rotation conditions is studied. Section 4.5 validates the effectiveness of the proposed algorithm with electromagnetic computational data, and the noise robustness of the proposed algorithm’s parameter estimation is evaluated.

**Figure 6:**
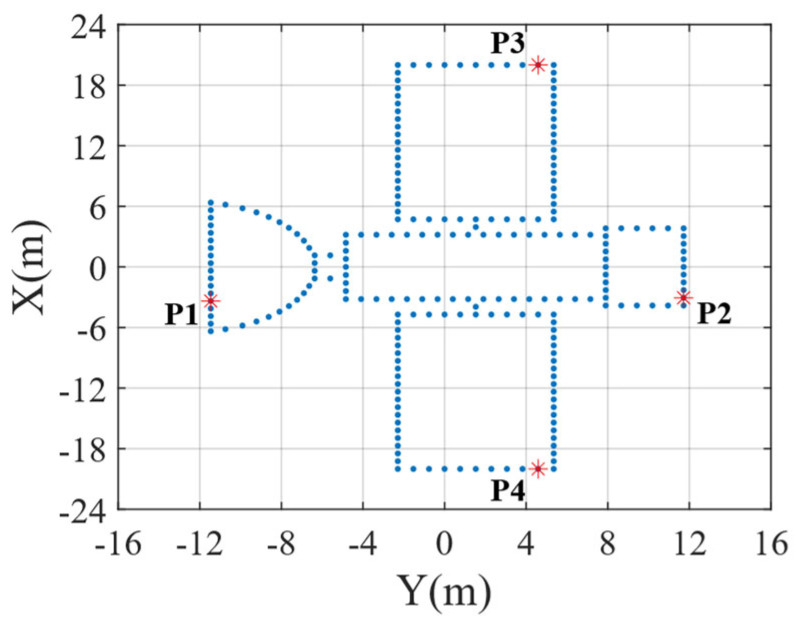
Scattering point target model. (Asterisks represent reference points.)

For Section 4.1, Section 4.2, Section 4.3 and Section 4.4, a target modeled with 235 scattering points was utilized to test the algorithm’s efficacy. The target structure is displayed in Figure 6, with a length of 23.185 m and a width of 40 m. In the target, four reference points are demarcated and labeled as P1, P2, P3, and P4. In the subsequent ISAR imaging scaling process, the distances between P1 and P2 and between P3 and P4 will be utilized to compute the scaling length and width for the target. It is assumed that the radar system transmits an LFM signal and adopts dechirping reception. The specific parameters of the radar system are outlined in Table 1. Unless otherwise stated, the radar echo signal-to-noise ratio (SNR) is 10 dB.

The simulation scenario satisfies the far-field conditions. Prior to imaging and scaling, the translational compensation has been completed, and the rotation center coincides with the scene center. The additional SCO is achieved by adding a constant phase term to the translation-compensated echoes. The LPFT [13] and PFA-EE [25] algorithms were selected as benchmarks. The LPFT algorithm, serving as a phase coefficient estimation method, commences by segmenting the ISAR image into sub-images based on amplitude intensity. It then optimizes the second-order phase coefficient for each sub-image by maximizing the image contrast (IC) and establishes a relationship between the range unit and the second-order phase coefficients. The RAV is computed from the slope of the line fitted via least squares, facilitating scaling. It is noteworthy that although the phase coefficient estimation method is inherently unsuitable for large-angle imaging scaling, the LPFT method’s sufficient robustness permitted its inclusion in specific simulation scenarios to demonstrate the characteristics of phase coefficient estimation methods. Moreover, the PFA-EE algorithm enhances image quality by exhaustively searching parameters for PFA imaging, utilizing the parameters that yield optimal imaging as an estimated result, thereby achieving scaling.

With respect to evaluation metrics, the non-uniform rotational parameters in this study include RAV and RAA. And, according to Equation (23), the scaling factors primarily depend on Φ. To conveniently evaluate scaling accuracy in extensive Monte Carlo experiments, the relative error (RE) of the estimated Φ is adopted as the evaluation metric. A smaller RE indicates higher scaling accuracy.

### 4.1. Simulations Under Varying Rotation Angles

**Figure 7:**
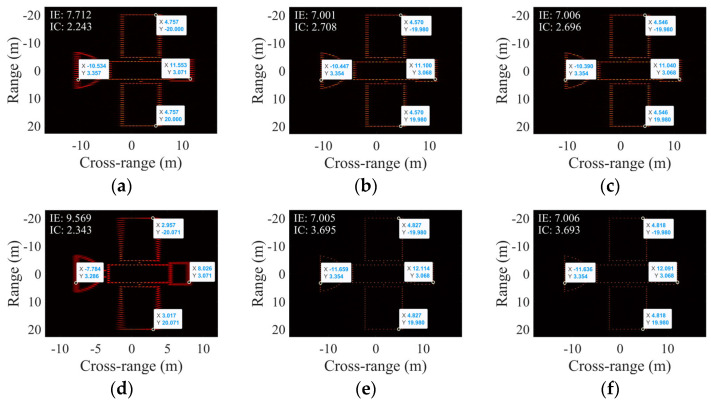
Cross-range scaling results under 1.5° for (**a**) LPFT, (**b**) PFA-EE, and (**c**) the proposed algorithm and under 6° for (**d**) LPFT, (**e**) PFA-EE, and (**f**) the proposed algorithm.

For this subsection, following translational motion compensation, the target was preset to rotate uniformly at an RAV of 0.0041 rad/s without SCO effects, and three sets of simulations were conducted at ERAs of 1.5°, 3°, and 6°. Figure 7 shows the ISAR results of different scaling algorithms at 1.5° and 6°, while Table 2 provides a statistical summary of the three sets of simulations.

In Figure 7, IE is displayed at each sub-figure’s top left, along with the coordinates of four reference points: P1, P2, P3, and P4. At the ERA of 1.5°, the imaging result of Figure 7a is marginally inferior to Figure 7c,d. This inferiority is attributed to the uncompensated quadratic term in LPFT used for estimating the RAV, which leads to the defocusing of partial scatterers. Nevertheless, the positions of the four reference points in these sub-figures of 1.5° are very close, and, alongside the values in Table 2, the estimated target lengths of the three algorithms are relatively consistent, with LPFT outperforming PFA-EE and the proposed algorithm. This suggests that the phase coefficient estimation method is effective for small rotation angles. As the ERA increases to 3° and 6°, both the imaging quality and cross-range scaling results of PFA-EE and the proposed algorithm surpass LPFT. This demonstrates the significant impact of $C_{oth}$ (see Equation (5)), which cannot be disregarded in a larger ERA. The estimation or compensation of each term within $C_{oth}$ would make the issue exceedingly complex, creating significant difficulty for the polynomial phase model. In contrast, the wave number domain signal model maintains a simplistic form, showcasing superior performance in imaging and scaling under large-angle rotation scenarios.

Moreover, Table 2 illustrates that regardless of the change in ERAs or the scaling algorithms, the estimated target width remains virtually unchanged. As the target’s width direction is parallel to the range direction, its scaling primarily depends on the radar system’s bandwidth and is nearly unaffected by the estimated rotation parameters. Consequently, the estimated target width will not be analyzed in subsequent simulation studies. From the analysis of the results in this subsection, LPFT was deemed unsuitable for scaling large-angle imaging and thus will no longer be compared in subsequent simulations.

### 4.2. Simulations with SCO

**Figure 8:**
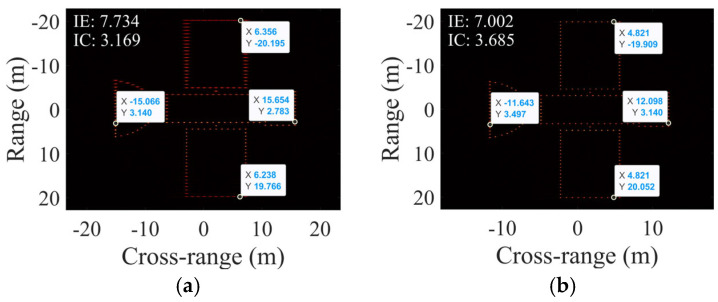
Cross-range scaling results under non-uniform rotation for (**a**) PFA-EE and (**b**) the proposed algorithm.

This subsection evaluates targets with uniform rotation (RAV: 0.0041 rad/s, ERA: 6°) and SCO = 5 m. Figure 8 compares the scaling results, with Table 3 listing numerical outcomes.

Figure 8a exhibits defocused scatterers compared to SCO-free results in Figure 7e, confirming SCO-induced degradation in PFA imaging. In contrast, the proposed method achieves focused imaging, as shown in Figure 8b, validating its SCO compensation capability.

Table 3 shows significant deviations in PFA-EE’s RAV and length estimation, while the proposed method maintains high accuracy in RAV, SCO, and length estimation, validating the effectiveness of the proposed scaling method under SCO conditions.

**Figure 9:**
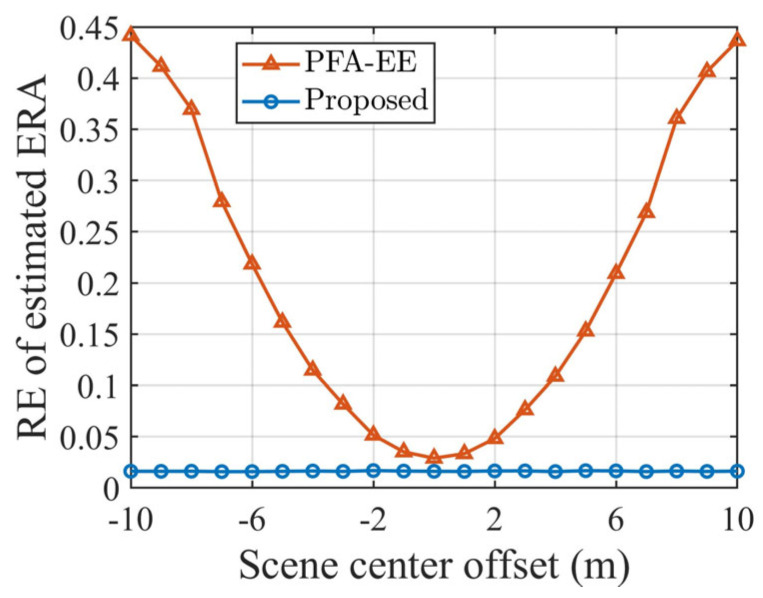
Cross-range scaling results under various scene center offsets for two algorithms.

To analyze SCO sensitivity, Monte Carlo experiments (50 trials per condition) were conducted with SCO varying from −10 m to 10 m (1 m intervals). The relative error of the ERA estimation is used as a metric for cross-range scaling. Figure 9 depicts the scaling results of the two algorithms under different SCOs.

In Figure 9, the scaling accuracy of PFA-EE deteriorates gradually as the SCO increases. Meanwhile, the proposed method’s scaling accuracy remains virtually unchanged, consistently staying below 0.05, further validating its effectiveness under SCO conditions.

### 4.3. Simulations with Non-Uniform Rotation

**Figure 10:**
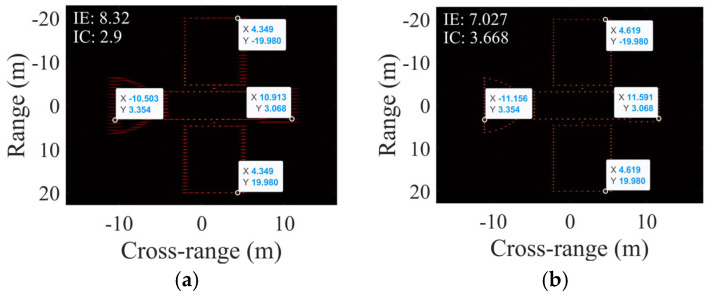
Cross-range scaling results with SCO for (**a**) PFA-EE and (**b**) the proposed algorithm.

This subsection examines the non-uniform target rotation scenario (RAV: 0.0041 rad/s, RAA: 2.9282 × 10^−5^ rad/s^2^, ERA: 6°, no SCO). Figure 10 shows scaling results, with Table 4 providing numerical comparisons.

Compared to Figure 7e, Figure 10a exhibits increased entropy and defocused scatterers, proving that non-uniform rotation degrades PFA imaging. This justifies the use of image quality optimization for estimating non-uniform rotation parameters in our study. In contrast, the proposed method still achieves focused imaging under non-uniform rotation conditions.

As shown in Table 4, PFA-EE’s estimates for RAV and the target length diverge from the true values, while the proposed method’s estimates for the RAV, RAA, and target length are all notably closer to true values, outperforming PFA-EE and confirming the effectiveness of the proposed method in non-uniform rotation conditions.

**Figure 11:**
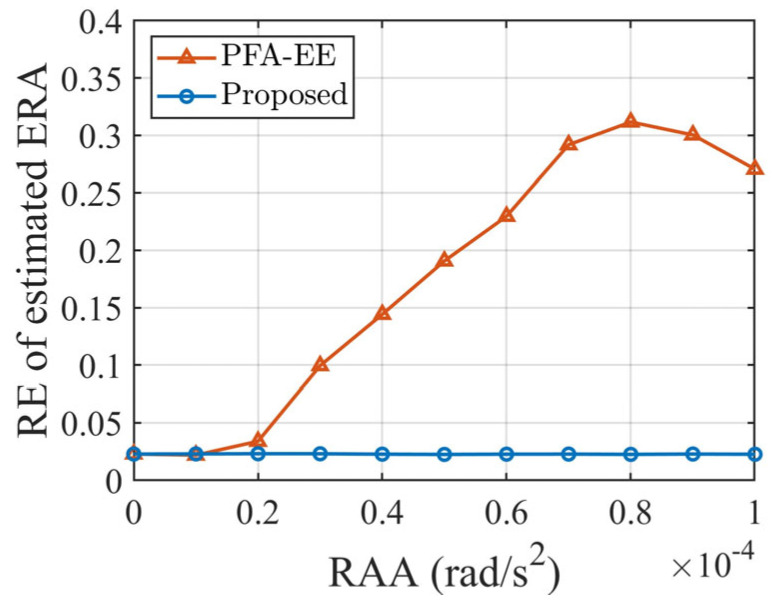
Cross-range scaling results under various RAAs for two algorithms.

To investigate the effect of the degree of non-uniform rotation on algorithm performance, the RAA value was changed while all other conditions were kept the same. The RAA values ranged from 1 × 10^−5^ rad/s^2^ to 1 × 10^−4^ rad/s^2^, with intervals of 1 × 10^−5^ rad/s^2^, and 50 Monte Carlo simulations were conducted for each simulation condition. Figure 11 demonstrates the cross-range scaling results of two algorithms under various RAAs.

As Figure 11 illustrates, the scaling accuracy of PFA-EE manifests a deteriorating trend with an increase in the RAA. Once the RAA surpasses 1 × 10^−5^ rad/s^2^, the effect of non-uniform rotation on the scaling precision becomes more pronounced. Conversely, the proposed method’s scaling accuracy remains relatively stable, always below 0.05, reinforcing its effectiveness under non-uniform rotation scenarios.

### 4.4. Simulations with Concurrent SCO and Non-Uniform Rotation

**Figure 12:**
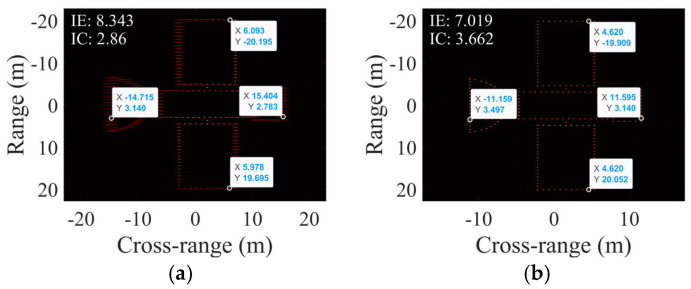
Cross-range scaling results with SCO and non-uniform rotation for (**a**) PFA-EE and (**b**) the proposed algorithm.

In this subsection’s simulations, non-uniform rotation of the target is accounted for, with an RAV of 0.0041 rad/s, RAA of 2.9282 × 10^−5^ rad/s^2^, ERA of 6°, and SCO of 5 m. Figure 12 presents the results of two scaling algorithms, while Table 5 provides the corresponding numerical metrics.

In Figure 12, partial scattering points in Figure 12a exhibit defocusing due to the inadequacy of the PFA under concurrent non-uniform rotation and SCO conditions. In comparison, the proposed method maintains focused imaging despite the combined effects of non-uniform rotation and SCO.

As shown in Table 5, PFA-EE’s estimates for RAV and target length substantially deviate from the true values, whereas the proposed method’s estimates for RAV, RAA, SCO, and target length are close to the true values, surpassing PFA-EE and verifying its effectiveness in composite scenarios.

Notably, as shown in Table 3 (SCO-only case), PFA-EE yields an RAV estimate of 0.0031 rad/s, while Table 5 (non-uniform rotation + SCO) reports 0.0032 rad/s. The estimation error for SCO decreased with the addition of non-uniform rotation, implicating that the effects of non-uniform rotation and SCO on PFA imaging are not merely linearly additive but complexly intercoupled. Nevertheless, the proposed algorithm still demonstrates robust parameter estimation accuracy under such coupled distortions.

### 4.5. Simulations with Electromagnetic Computational Data

**Figure 13:**
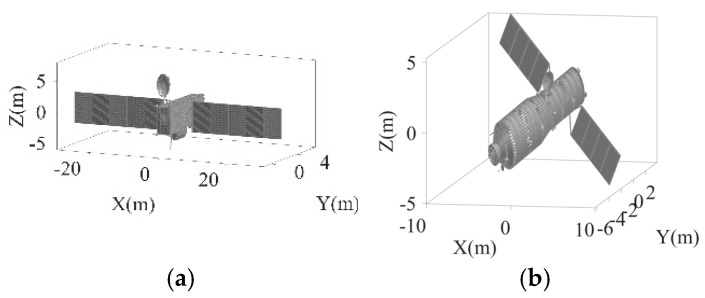
Electromagnetic computing target model. (**a**) Target 1. (**b**) Target 2.

**Figure 14:**
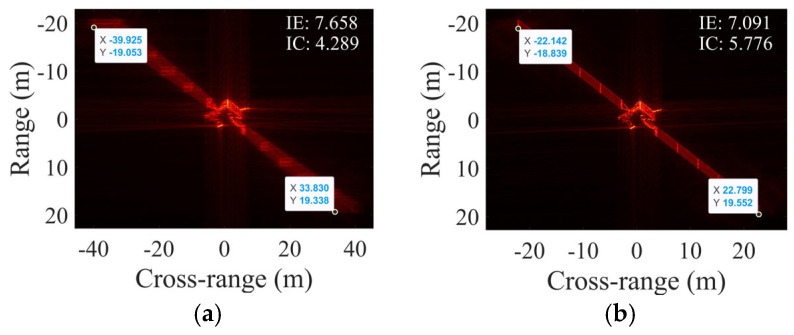

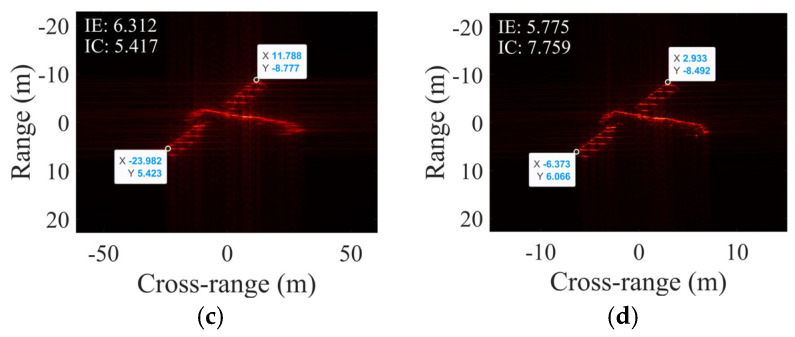
Cross-range scaling results of electromagnetic computing data. Target 1 with (**a**) PFA-EE and (**b**) proposed algorithm. Target 2 with (**c**) PFA-EE and (**d**) proposed algorithm.

This subsection verifies the effectiveness of the proposed algorithm using electromagnetic computational data. The target exhibits a non-uniform rotation characterized by an RAV of 0.0041 rad/s, an RAA of 2.9282 × 10^−5^ rad/s^2^, and an ERA of 6°, with SCO = 5 m. The electromagnetic computational data were obtained with the Physical Optics (PO) method [34]. The facet models of two targets are depicted in Figure 13. Target 1 has a length of 60 m, a width of 8 m, and an approximate height of 15 m. At the zero moment of imaging, the target’s pitch angle is 24.268°, and its azimuthal angle is 45.160°. As for target 2, it has a length of 19.21 m, a width of 10.41 m, and a height of 3.55 m. Its pitch angle and azimuthal angle are −45° and −60°, respectively, at the zero moment of imaging. Figure 14 illustrates the scaled images of two targets with two algorithms, and Table 6 provides the corresponding numerical results.

As shown in Figure 14, PFA-EE exhibits defocusing attributed to its disregard of RAA and SCO, while the proposed method achieves focused imaging. The target length in the ISAR image is calculated using two points on the target’s edge.

As shown in Table 6, the estimated RAV of PFA-EE deviates from the true value. In contrast, the proposed algorithm yields RAV, RAA, and SCO estimates that are near the true values. According to the target’s initial posture, based on the target’s initial posture and scaling information in Figure 14, the estimated length of targets can be calculated. Furthermore, the RE of target length estimation can be obtained. The proposed algorithm demonstrates significantly lower RE in length estimation for different targets compared to PFA-EE, confirming its superior effectiveness.

**Figure 15:**
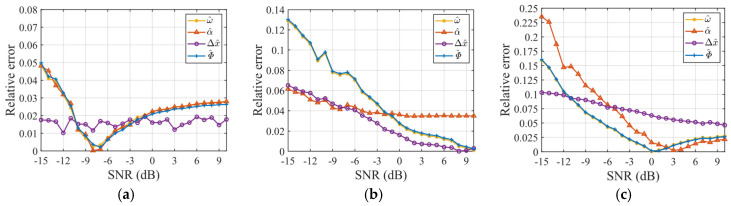
Relative error in parameter estimation for three targets under various SNRs. (**a**) Scattering point target in Figure 6. (**b**) Electromagnetic computing target 1. (**c**) Electromagnetic computing target 2.

To extend the evaluation of the noise robustness of the proposed algorithm, simulations were repeated under varying SNRs from −15 dB to 10 dB (1 dB intervals), with 50 Monte Carlo trials per SNR. Figure 15 shows three targets’ relative errors of the estimated RAV ($\hat{\omega }$), RAA ($\hat{\alpha }$), SCO ($△\hat{x}$), and ERA ($\hat{\Phi }$) under different SNRs.

**Figure 16:**
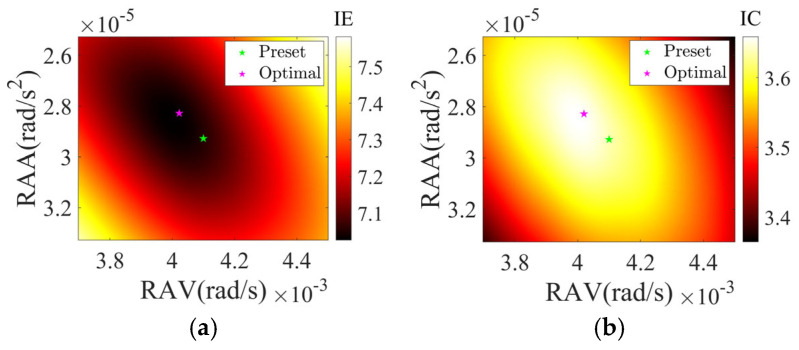
(**a**) IE and (**b**) IC of ISAR imaging results under different rotational parameters.

As shown in Figure 15, the overall relative error level of the scattering point target is lower than the other two electromagnetic calculation targets. This is because the latter have more complex electromagnetic scattering characteristics compared to ideal point targets, leading to algorithm performance degradation. Notably, as shown in Figure 15a,c, as the SNR increases, the relative error of rotational parameter estimation initially decreases and then increases. This unusual phenomenon was investigated by calculating the IE and IC values of the scattering point target imaging result near preset rotational parameters under noiseless and SCO-free conditions, with visualization results shown in Figure 16. The optimal point corresponds to rotational parameters when IE is minimized or IC is maximized. The results demonstrate deviations between the optimal and the preset point, indicating the occurrence of estimation biases when IE or IC is used as an image quality metric for parameter optimization. These target-dependent biases contribute to the differing SNR curves for various targets in Figure 15. Moreover, estimation biases may also relate to parameter coupling, target motion, or noise, which are complex and common issues in ISAR imaging and parameter estimation, and this study will not elaborate further.

**Figure 17:**
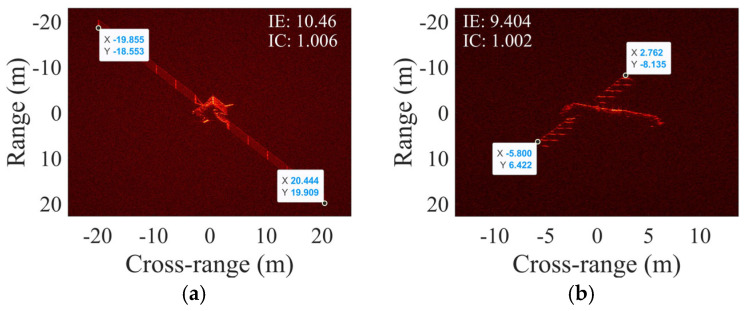
Cross-range scaling results of electromagnetic computing data under −5 dB for (**a**) target 1 and (**b**) target 2.

Despite the estimator biases, Figure 15 shows that when the SNR exceeds −5 dB, the proposed algorithm maintains rotational parameter estimation errors below 0.05 for all targets. The parameter estimation performance of the proposed algorithm is reliable under this condition. Furthermore, Figure 17 and Table 7 present the scaling results of two electromagnetic targets at −5 dB. The relative errors of the estimated length are below 0.03, achieving excellent scaling accuracy.

## 5. Conclusions

To address the increased cross-range scaling error in large-angle ISAR imaging caused by SCO and non-uniform target rotation, this paper presents a novel wavenumber-domain scaling method. By analyzing the limitations of the traditional polynomial phase history model, a more concise and accurate signal model suitable for large-angle ISAR imaging was developed. By integrating PSO search and IE optimization, a joint estimation algorithm for non-uniform rotation parameters and SCO was proposed, enabling the precise estimation of unknown parameters. Post-SCO compensation and wavenumber-domain processing with the estimated parameters facilitate focused imaging. Furthermore, employing the derived scaling factor achieves accurate scaling of large-angle ISAR images with non-uniform rotation. The proposed algorithm’s scaling precision is comparable to that of the PFA for uniform rotation and surpasses the PFA when factoring in SCO and non-uniform rotation. With an SNR of −5 dB, the rotation parameter estimation error of the proposed algorithm is less than 0.05, and the relative scaling error for electromagnetic calculation data targets remains below 0.03. In the future, we will conduct more in-depth research on the estimator biases inherent in IE and IC as image quality optimization metrics and explore approaches for reducing the computational complexity of the algorithm.

## Figures and Tables

**Table 1:** Radar system parameters.

Parameters	Values
Carrier frequency	16.1 GHz
Bandwidth	2 GHz
Pulse width	200 μs
Dechirping pulse width	210 μs
PRF	40 Hz

**Table 2:** Numerical results under 1.5°, 3°, and 6° by different cross-range algorithms.

	ERA	Estimated RAV (rad/s)	Estimated Length (m)	RE of Estimated Length	Estimated Width (m)	RE of Estimated Width
	1.5°	0.00425	22.089	0.0548	40	0
LPFT	3°	0.00573	16.65	0.3267	40.142	0.0071
	6°	0.00603	15.812	0.369	40.142	0.0071
	1.5°	0.00444	21.559	0.0813	39.96	0.002
PFA-EE	3°	0.00399	23.943	0.0379	39.96	0.002
	6°	0.004	23.775	0.295	39.96	0.002
	1.5°	0.00443	21.432	0.0877	39.96	0.002
Proposed algorithm	3°	0.00399	23.846	0.0331	39.96	0.002
	6°	0.004	23.729	0.272	39.96	0.002

**Table 3:** Numerical results in Figure 8.

	Estimated RAV (rad/s)	Estimated SCO (m)	Estimated Length (m)	RE of Estimated Length
PFA-EE	0.0031	-	30.722	0.377
Proposed algorithm	0.004	4.901	23.744	0.028

**Table 4:** Numerical results in Figure 10.

	Estimated RAV (rad/s)	Estimated RAA (rad/s^2^)	Estimated Length (m)	RE of Estimated Length
PFA-EE	0.00444	-	21.418	0.0883
Proposed algorithm	0.004	2.85 × 10^−5^	22.749	0.0218

**Table 5:** Numerical results in Figure 12.

	Estimated RAV (rad/s)	Estimated RAA (rad/s^2^)	Estimated SCO (m)	Estimated Length (m)	RE of Estimated Length
PFA-EE	0.0032	-	-	30.121	0.347
Proposed algorithm	0.004	2.85 × 10^−5^	4.9	22.757	0.0214

**Table 6:** Numerical results in Figure 14.

	Target	Estimated RAV (rad/s)	Estimated RAA (rad/s^2^)	Estimated SCO (m)	Estimated Length (m)	RE of Estimated Length
PFA-EE	1	0.00239	-	-	86.877	0.4479
Proposed algorithm	1	0.0041	2.82 × 10^−5^	5.0446	59.106	0.0293
PFA-EE	2	8.963 × 10^−4^	-	-	43.734	1.277
Proposed algorithm	2	0.00392	2.77 × 10^−5^	4.951	19.641	0.0221

**Table 7:** Numerical results in Figure 17.

	Estimated RAV (rad/s)	Estimated RAA (rad/s^2^)	Estimated SCO (m)	Estimated Length (m)	RE of Estimated Length
Target 1	0.0045	2.754 × 10^−5^	4.741	58.206	0.0299
Target 2	0.00425	3.236 × 10^−5^	4.572	19.191	9.891 × 10^−4^

## Data Availability

Data are available on request from the authors.

## Appendix A

In this part, we present a detailed derivation from Equations (16) and (17). Initially, the 2D-DIFT of Equation (16) is expressed as follows:

(A1) $$\begin{array}{l} s_{\mathrm{Img},p}(k_{x},k_{y})\\ =\sigma (x_{p},y_{p})\frac{1}{N_{x}}\sum_{n_{x}=0}^{N_{x}-1}\;rect\left(\frac{n_{x}△f_{x}-2f_{c}/c}{N_{x}△f_{x}}\right)\exp\left(-j2\pi x_{p}n_{x}△f_{x}\right)\;\exp\left(j2\pi \frac{n_{x}k_{x}}{N_{x}}\right)\;\;\;\frac{1}{N_{y}}\;\sum_{n_{y}=0}^{N_{y}-1}rect\left(\frac{n_{y}}{N_{y}}\right)\exp\left(-j2\pi y_{p}n_{y}△f_{y}\right)\exp\left(j2\pi \frac{n_{y}k_{y}}{N_{y}}\right)\\ \;\;\;\;=\sigma (x_{p},y_{p})\frac{1}{N_{x}}\sum_{n_{x}=0}^{N_{x}-1}\;rect\left(\frac{n_{x}△f_{x}-2f_{c}/c}{N_{x}△f_{x}}\right)\exp\left(-j2\pi x_{p}n_{x}△f_{x}\right)\;\exp\left(j2\pi \frac{n_{x}k_{x}}{N_{x}}\right)\;\;\cdot \mathrm{DIFT}\left[rect\left(\frac{n_{y}}{N_{y}}\right)\exp\left(-j2\pi y_{p}n_{y}△f_{y}\right)\right]. \end{array}$$

The DIFT part in Equation (A1) is analyzed, and the DIFT of the rectangular function is determined to satisfy

(A2) $$\mathrm{DIFT}\left[rect\left(\frac{n_{y}}{N_{y}}\right)\right]=\frac{\sin(\pi k_{y})}{N\sin(\pi k_{y}/N_{y})}.$$

Leveraging the time-shifting property of the DIFT, it can be subsequently derived that

(A3) $$\mathrm{DIFT}\left[rect\left(\frac{n_{y}}{N_{y}}\right)\exp\left(-j2\pi y_{p}n_{y}△f_{y}\right)\right]=\frac{\sin\left(\pi \left(k_{y}-y_{p}N_{y}△f_{y}\right)\right)}{N_{y}\sin\left(\pi \left(k_{y}-y_{p}N_{y}△f_{y}\right)/N_{y}\right)}.$$

Equation (A3) is substituted into Equation (A1), resulting in

(A4) $$\begin{array}{c} s_{Img,p}(k_{x},k_{y})=\underset{x\to \infty }{\lim}\sigma (x_{p},y_{p})\frac{1}{N_{x}}\sum_{n_{x}=0}^{N_{x}-1}\;rect\left(\frac{n_{x}△f_{x}-2f_{c}/c}{N_{x}△f_{x}}\right)\exp\left(-j2\pi x_{p}n_{x}△f_{x}\right)\;\exp\left(j2\pi \frac{n_{x}k_{x}}{N_{x}}\right)\frac{\sin\left(\pi \left(k_{y}-y_{p}N_{y}△f_{y}\right)\right)}{N_{y}\sin\left(\pi \left(k_{y}-y_{p}N_{y}△f_{y}\right)/N_{y}\right)}\\ \;\;\;\;\;\;=\sigma (x_{p},y_{p})\frac{\sin\left(\pi \left(k_{y}-y_{p}N_{y}△f_{y}\right)\right)}{N_{y}\sin\left(\pi \left(k_{y}-y_{p}N_{y}△f_{y}\right)/N_{y}\right)}\mathrm{DIFT}\left[rect\left(\frac{n_{x}△f_{x}-2f_{c}/c}{N_{x}△f_{x}}\right)\exp\left(-j2\pi x_{p}n_{x}△f_{x}\right)\right]. \end{array}$$

We consider the DIFT part in Equation (A4).

(A5) $$\begin{array}{c} \mathrm{DIFT}\left[rect\left(\frac{n_{x}△f_{x}-2f_{c}/c}{N_{x}△f_{x}}\right)\exp\left(-j2\pi x_{p}n_{x}△f_{x}\right)\right]=\mathrm{DIFT}\left[rect(\frac{n_{x}-\frac{2f_{c}}{c△f}}{N_{x}})\exp\left[-j2\pi \left(x_{p}\left(n_{x}-\frac{2f_{c}}{c△f}\right)△f_{x}\right)\right]\exp\left(-j4\pi \frac{x_{p}f_{c}}{c}\right)\right]\\ \;\;\;\;\;\;\;\;\;\;\;\;\;\;\;\;\;\;\;\;=\frac{\sin\left(\pi \left(k_{x}-x_{p}N_{x}△f_{x}\right)\right)}{N_{x}\sin\left(\pi \left(k_{x}-x_{p}N_{x}△f_{x}\right)/N_{x}\right)}\exp\left(j4\pi \frac{k_{x}f_{c}}{cN_{x}△f_{x}}\right)\exp\left(-j4\pi \frac{x_{p}f_{c}}{c}\right). \end{array}$$

By substituting Equation (A5) into Equation (A4) and simplifying, the following result is obtained:

(A6) $$s_{Img,p}(k_{x},k_{y})=\frac{\sin\left(\pi \left(k_{x}-2x_{p}F_{x}\right)\right)}{N_{x}\sin\left(\pi \left(k_{x}-2x_{p}F_{x}\right)/N_{x}\right)}\frac{\sin\left(\pi \left(k_{y}-2y_{p}F_{y}\right)\right)}{N_{y}\sin\left(\pi \left(k_{y}-2y_{p}F_{y}\right)/N_{y}\right)}\exp\left[j2\pi \frac{k_{x}f_{c}}{cF_{x}}\right]\exp\left[-j4\pi \frac{x_{p}f_{c}}{c}\right]\sigma (x_{p},y_{p})$$

Finally, Equation (A6) corresponds to Equation (17) in the main text.
